# Improving the regenerative potential of olfactory ensheathing cells by overexpressing prostacyclin synthetase and its application in spinal cord repair

**DOI:** 10.1186/s12929-017-0340-1

**Published:** 2017-05-25

**Authors:** May-Jywan Tsai, Chi-Ting Huang, Yong-San Huang, Ching-Feng Weng, Song-Kun Shyue, Ming-Chao Huang, Dann-Ying Liou, Yan-Ru Lin, Chu-Hsun Cheng, Huai-Sheng Kuo, Yilo Lin, Meng-Jen Lee, Wen-Hung Huang, Wen-Cheng Huang, Henrich Cheng

**Affiliations:** 1 Neural Regeneration Laboratory, Center for Neural Regeneration, Department of Neurosurgery, Neurological Institute, Taipei Veterans General Hospital, No. 322, Section 2, Shih-Pai Road, Beitou District, Taipei, 11217 Taiwan; 2 Department of Veterinary Medicine, College of Veterinary Medicine, National Chung Hsing University, Taichung, 40227 Taiwan; 3 Institute of Biotechnology, National Dong Hwa University, Hualien, 97401 Taiwan; 4 Institute of Biomedical Sciences, Academia Sinica, Taipei, 11529 Taiwan; 5 Center for Neural Regeneration, Neurological Institute, Taipei Veterans General Hospital, Taipei, 11217 Taiwan; 6 Graduate Institute of Veterinary Pathobiology, College of Veterinary Medicine, National Chung Hsing University, Taichung, 40227 Taiwan; 7 Department of Applied Chemistry, Chaoyang University of Technology, Taichung, 41349 Taiwan; 8 Institute and Department of Pharmacology, National Yang-Ming University, Taipei, 11221 Taiwan

**Keywords:** Prostacyclin, Gene transfer, Transplantation, Spinal nerve regeneration, Olfactory ensheathing glia

## Abstract

**Background:**

Olfactory ensheathing cells (OEC), specialized glia that ensheathe bundles of olfactory nerves, have been reported as a favorable substrate for axonal regeneration. Grafting OEC to injured spinal cord appears to facilitate axonal regeneration although the functional recovery is limited. In an attempt to improve the growth-promoting properties of OEC, we transduced prostacyclin synthase (PGIS) to OEC via adenoviral (Ad) gene transfer and examined the effect of OEC with enhanced prostacyclin synthesis in co-culture and in vivo. Prostacyclin is a vasodilator, platelet anti-aggregatory and cytoprotective agent.

**Results:**

Cultured OEC expressed high level of cyclooxygneases, but not PGIS. Infection of AdPGIS to OEC could selectively augument prostacyclin synthesis. When cocultured with either OEC or AdPGIS-OEC, neuronal cells were resistant to OGD-induced damage. The resulted OEC were further transplanted to the transected cavity of thoracic spinal cord injured (SCI) rats. By 6 weeks post-surgery, significant functional recovery in hind limbs occurred in OEC or AdPGIS-OEC transplanted SCI rats compared with nontreated SCI rats. At 10–12 weeks postgraft, AdPGIS-OEC transplanted SCI rats showed significantly better motor restoration than OEC transplanted SCI rats. Futhermore, regenerating fiber tracts in the distal spinal cord stump were found in 40–60% of AdPGIS-OEC transplanted SCI rats.

**Conclusions:**

Enhanced synthesis of prostacyclin in grafted OEC improved fiber tract regeneration and functional restoration in spinal cord injured rats. These results suggest an important potential of prostacyclin in stimulating OEC therapeutic properties that are relevant for neural transplant therapies.

## Background

Complete transection of the adult mammalian spinal cord results in irreversible and permanent loss of motor and somatosensory function below the injury site [1–3]. The inhospitable environment and lack of spontaneous regeneration of injured central axons lead to permanent functional impairment in the adult SCI victims [4–6]. However, this does not apply to the olfactory bulb. The growth permissive olfactory bulbs are due to the presence of olfactory ensheathing cells (OEC), the distinct glia of the primary olfactory nerve with both Schwann cell-like and astrocyte-like characteristics [7–10]. The presence of OEC is thought to provide support and allow growing axons of olfactory sensory neurons to cross the transition zone between PNS and CNS and extend processes in the olfactory bulb without inhibition [9–12]. Several groups have used OEC transplant to stimulate axonal elongation in different injured CNS regions [13–17]. After implantation in the spinal cord, OEC induced a less severe host astrocyte response than Schwann cells [18]. Thus, OEC transplantation appears to be a promising treatment for spinal cord injury, although the improvement of functional recovery is limited [14, 19, 20]. Overexpression of neuroprotective genes seems a promising strategy to promote regeneration of injured axon. In an attempt to further increase the growth promoting effect of OEC, the present study employs ex vivo adenovirus-mediated gene transfer of protacyclin synthase (PGIS) in OEC for enhancing prostacyclin synthesis.

Prostacyclin (prostaglandin (PG) I_2_) acts as a primary vasodilator and is an inhibitor of leukocyte adhesion and platelet aggregation [21, 22]. PGI_2_ is synthesized by the sequential action of cyclooxygenase (COX)-1 or −2 and prostacyclin synthase from arachidonic acid (AA) which is deacylated from cellular phospholipids [23]. Prostaglandin or leukotriene were rapidly produced following tissue injury or inflammation [24]. Prostacyclin or its CNS analogs have been reported to reduce infarct volume in ischemic rat brains [25, 26]. Administration of prostacyclin significantly reduced the cortical lesion in rats with traumatic brain injury [27]. Lin et al., [25] and our recent articles [28, 29] have demonstrated beneficial effects of enhanced prostacyclin synthesis in neuronal cultures as well as in Parkinsonian or ischemic brains. In multiple sclerosis, vessels-derived prostacyclin could facilitate axonal remodeling of injured neuronal networks and promote sprouting of CST fibers, contributing to the repair process [30]. Prostacyclin could promote remyelination and functional recovery in mice after spinal nerve demyelination [31]. These findings prompted us to test the possibility that prostacyclin contributes to spinal nerve regeneration. We have constructed recombinant adenovirus vectors (Ad) encoding green fluorescence protein (GFP), COX-1, COX-2 or PGIS, allowing for stable delivery of individual gene in infected cells. By combinational infection of AdCOX-1 and/or AdPGIS, we have selectively enhanced prostacyclin production in endothelial cells [32, 33] or in CNS cells [28, 29]. The present work aims to assess the effects of augmented prostacyclin production in OEC by adenoviral gene transfter on survival and regeneration of spinal axons after transplantation of AdPGIS-OEC to thoracic T8-transected spinal cord. Our goal is to provide an environment more conducive to spinal nerve regrowth after spinal cord injury.

## Methods

### Reagents and antibodies

Cultured medium, fetal bovine serum (FBS), serum-free supplements and antibiotics were purchased from Invitrogen (Carlsbad, CA, USA). Tissue culture plastics were from BD Bioscience (San Jose, CA, USA). Millicell culture inserts were from Millipore (Watford, UK). Primary antibodies used in the present study were: rabbit or mouse antineuronal class III beta tubulin (clone TUJ-1, Covance, NJ, USA), rabbit anti-GFAP or anti-S100 (Dako Cytomation, Ely, UK), mouse anti-low affinity nerve growth factor receptor (p75^NGFR^)(anti-P75),rabbit anti-serotonin (Chemicon) and goat anti-β-actin (Santa Cruz Biotechnology, Texas, USA). Unless stated otherwise, all other chemicals were purchased from Sigma-Aldrich Co.

### Cell culture and in vitro transduction

The procedure to culture and purify OEC followed the method described in Nash et al., [34] with modification. Briefly, the outer two layers of the adult Spraque-Dawley (SD) rat olfactory bulbs were dissected and cleaned of all meninges and blood vessels. The tissues were minced, triturated and trypsinized. After removal of trypsin, the dissociated cells were seeded to uncoated dishes and maintained in DMEM/F-12 (1:1 mixture, Invitrogen) supplemented with 10% FBS for 48 h (at 37 °C and 5% CO_2_). The suspended cells were then collected, reseeded to poly-D-lysine-coated dishes and maintained in DMEM/F12 supplemented with 10% FBS and astrocyte-conditioned medium (ACM). Astrocytes were prepared as described in our recent articles [29, 35]. ACM were collected from the released medium of confluent astrocytes after conditioning for 2 days. OEC cultures were refilled with fresh medium and ACM every 2 days until they reached confluence. Confluent OEC were subcultured for immunohistochemistry or for co-culture studies (by seeding to insert well). For in vivo study, confluent OEC were infected with AdGFP or AdPGIS 2–3 days before processing for measuring activity to synthesize eicosanoids from AA or for transplantation to SCI rats.

### Neuronal cultures and OGD treatment

Mixed neuron-glial cell cultures were prepared from the cortical or spinal cord regions of Spraque-Dawley (SD) rat fetus at gestation of 15–17 days as described in Tsai et al. [36, 37]. Briefly, cells were dissociated with mixtures of papain/protease/deoxyribonuclease I (0.1%:0.1%:0.03%) and plated onto poly-D- lysine-coated dishes in DMEM supplemented with 10% FBS. The cells were incubated at 37 °C in a water-saturated atmosphere of 5% CO_2_/95% air. For OGD treatment, the culture media were replaced with glucose-free DMEM and placed into an air-tight chamber to obtain 0.5% O_2_ with a gas mixture 95% N_2_/5% CO_2_. After 3 h of OGD, reperfusion was simulated by replacing the exposure medium with normal growth medium. Cultures were then co-cultured with none, OEC or AdPGIS-OEC at 0 h after OGD reperfusion. Two days later, cultures were treated with propidium iodide (PI, 1 μg/ml) for 4 h before fixation for immunohistochemistry. Images of immunoreactive (IR) or PI (+) cells were obtained under a fluorescent microscope equipped with a cooling digital imaging system. BetaIII tubulin-IR neurite density and PI (+) or ED1 (+) cell numbers (obtained using a 20x object lens) were analyzed using Image-Pro plus software (NIH systems, Bethesda, MD, USA).

### Recombinant adenovirus (Ad)

Replication-defective first generation E1-deleted adenoviral vectors were used. The recombinant adenoviruses used phosphoglycerate kinase (PGK) as a driving promoter. AdPGK (containing the promoter only without encoding gene) was used as a vector control. AdGFP was utilized for optimizing the infection conditions. Large-scale production of high titer (∼10^10^ pfu/mL) of adenovirus encoding none, GFP, or PGIS was performed as described in previous articles [28, 29]. AdGFP was added to OEC culture with a multiplicity of injection (MOI) of 50, resulting in transduction of virtually all cultured cells. Thus, 50 MOI each of recombinant Ads was used for infection to cultured OEC ex vivo. OEC were grafted to SCI rats at 2–3 days after being infected with AdPGK, AdGFP or AdPGIS ex vivo.

### Extraction and analysis of arachidonic acid (AA) metabolites in cultured cells

Cultured OEC after recombinant adenoviral infection for 2 – 3 days was measured for their activity to synthesize eicosanoids from AA according to the methods described in our recent articles [28, 29]. Briefly, confluent cells were incubated in DMEM/F12 (serum-free) containing 10 μM [1-^14^C] AA at 37 °C for 10 min. The resulted cells were harvested for western blot analysis and the media, containing released eicosanoids, were saved for analysis of [1-^14^C] labeled AA metabolites. The extracted [1-^14^C] labeled AA metabolites were analyzed by reverse phase HPLC, a solvent delivery system (Waters model 2690) equipped with an on-line radioisotopic detector (Packard 150-TP) followed methods described previously [29, 32]. The [1-^14^C] eicosanoids were identified by their retention times with the authentic radioisotopic standands.

### Animal surgery and treatment

Adult SD rats, obtained from NYU Animal Breeding and Research Center in Taiwan, were used for the present study. The surgical procedures for spinal cord transection and post-operative animal care had been reviewed and approved by the Institutional Animal Care and Use Committee (IACUC) of Taipei Veterans General Hospital and Reg. No 2014–002. All efforts were taken to minimize animal suffering during and following surgery and to reduce the numbers of animals that were sacrificed. Spinal cord transection surgery was performed according to the previously described protocols [36, 38, 39]. Briefly, rats were anesthetized with 1 – 2% isoflurane (Baxter, Guayama, PR, USA). The body temperature was maintained during surgery at 37 ± 0.5 °C with a heating pad servo-controlled by a rectal probe. A laminectomy was made at the thoracic vertebral level 8 (T8). The meningeal membranes were severed along with the dura mater. The spinal cord was completely transected with a 5 mm segment of spinal cord encompassing T8 being removed. The rostral and caudal stumps were lifted after removal of the spinal cord segment to ensure complete discontinuity. Transplanted cells, a total of 5× 10^5^ OEC in fibrin solution, were applied to the transected cavity. The muscle layers and the skins were closed separately. Bladder evacuation was applied in experimental rats twice daily. Behavior test for hindlimb performance were conducted biweekly after transplantation. At 3 months postgrafting, experimental rats were processed for anterograde tracing or for immunohistochemical staining.

### Histological procedures

Anterograde axonal tracing using WGA-horseradish peroxidase (HRP) was conducted in experimental rats at 12 week after surgery and transplantation. WGA-HRP was injected stereotaxically into the hindlimb area of the sensory motor cortex (10 sites, bilaterally, 0.3 μl/site) in experimental rats under anesthesia. All injections were performed over a 3 min period, and the injection needle was kept in place for an additional 3 min to minimize leakage on needle withdrawal. After the injection, the muscles and skin were closed in layers. Rats were further kept for 2 days before processing for tracer identification in spinal cords. Briefly, longitudinal sections (14 μm) of thoracic spinal cord (T7-T9) were incubated with tetramethylbenzidine (TMB) for HRP histochemistry according to TMB substrate kit (SK-4400, Vector). For immunohistochemical analysis, rats were anesthetized and transcardically perfused with phosphate-buffered 4% paraformaldehyde at 3 or 12 weeks after transplanation. The spinal segments were postfixed in 4% paraformaldehyde overnight, and then cryoprotected in PBS containing 30% (w/v) sucrose for 3 days. The tissues were excised and embedded in Tissue TekOCT (Sakura Fine Technical, Tokyo, Japan) and then cross-sectioned at 20 $\mu$m thickness with a cryostat.

### Immunohistochemical analysis

Frozen spinal sections were processed for immunohistochemistry followed method described in our recent articles [35, 39]. The tissue sections were incubated with primary antibodies, followed by respective 2nd antibodies for histological evaluation as described. Primary antibodies used were: GFP (1:200, Millipore, Billerica, MA), iba1(1:400, Abcam, Cambridge, UK), ED1 (1:250, AbD Serotec, UK), GFAP (1:400; DakoCytomation,Glostrup,Denmark), S100 beta (1:250; Sigma), p75^NGFR^(+) (1:500; Millipore, Billerica, MA) and type III beta tubulin (1:250; Covance Research Products Inc., Denver, PA). For double immunostaining, the secondary antibodies used were Alex 488 fluorophore donkey anti-rabbit antibody (1:200; Molecular Probes, Eugene, OR, USA) and Cy3-conjugated donkey anti-mouse antibody (1:200; Jackson ImmunoResearch Laboratories, West Grove, PA). Primary antibody omission controls were performed for all immunostaining protocols to control for nonspecific binding. Fluorescent visualization and photography were performed on a Zeiss Axioscope microscope with appropriate filter sets (Zeiss, Oberkochen, Germany).

### Behavioral assessment

Hindlimb performance was evaluated using the open field locomotor test initially developed by Basso et al. [40] as described in our recent articles [36, 39]. Two observers, unaware of the experimental procedures, performed the test biweekly according to the Basso Beattie Bresnahan (BBB) open field locomotor test and Combined Behavior Score (CBS) scales. The BBB scale ranges from 0 (no hindlimb movement) to 21 (normal movement-coordinated gait) [40].

### Western blot analysis

After experimental periods, cultured cells were washed twice with PBS and solubilized in lysis buffer containing 40 mM Tris buffer (pH 7.5), 8 M urea, 4% CHAPS, 1 mM PMSF, 1 mM Na_3_VO_4_, 1 mM dithiothreitol, and a protease inhibitor kit (BM, Germany). Protein was quantified by a Biorad protein assay reagent. Aliquots of proteins (5 μg) of the cell lysates were analyzed by western blot, using SDS-PAGE (8% or 12% gel), as previously described [35, 41]. After electrophoresis, gels were transferred to PVDF membranes (Millipore Corp., USA) and incubated overnight at 4 °C with antibodies against PGIS (rabbit, dilution 1/3000), COX-1, COX-2 (rabbit, dilution 1/5000, Cayman) or β-actin (1:5000; Santa Cruz Biotech, USA) followed by a HRP-conjugated secondary antibody (dilution 1/2000, Jackson Lab) for 1 h at room temperature. Immunoreactivity was visualized by enhanced chemiluminescent detection (Perkin Elmer Co, USA).

### Statistical analysis

Experimental data were expressed as the mean of independent values ± s.e.m. and were analyzed using one-way analysis of variance (ANOVA) followed by LSD test. Values of *P* < 0.05 were considered to show statistical significance.

## Results

**Fig. 1:**
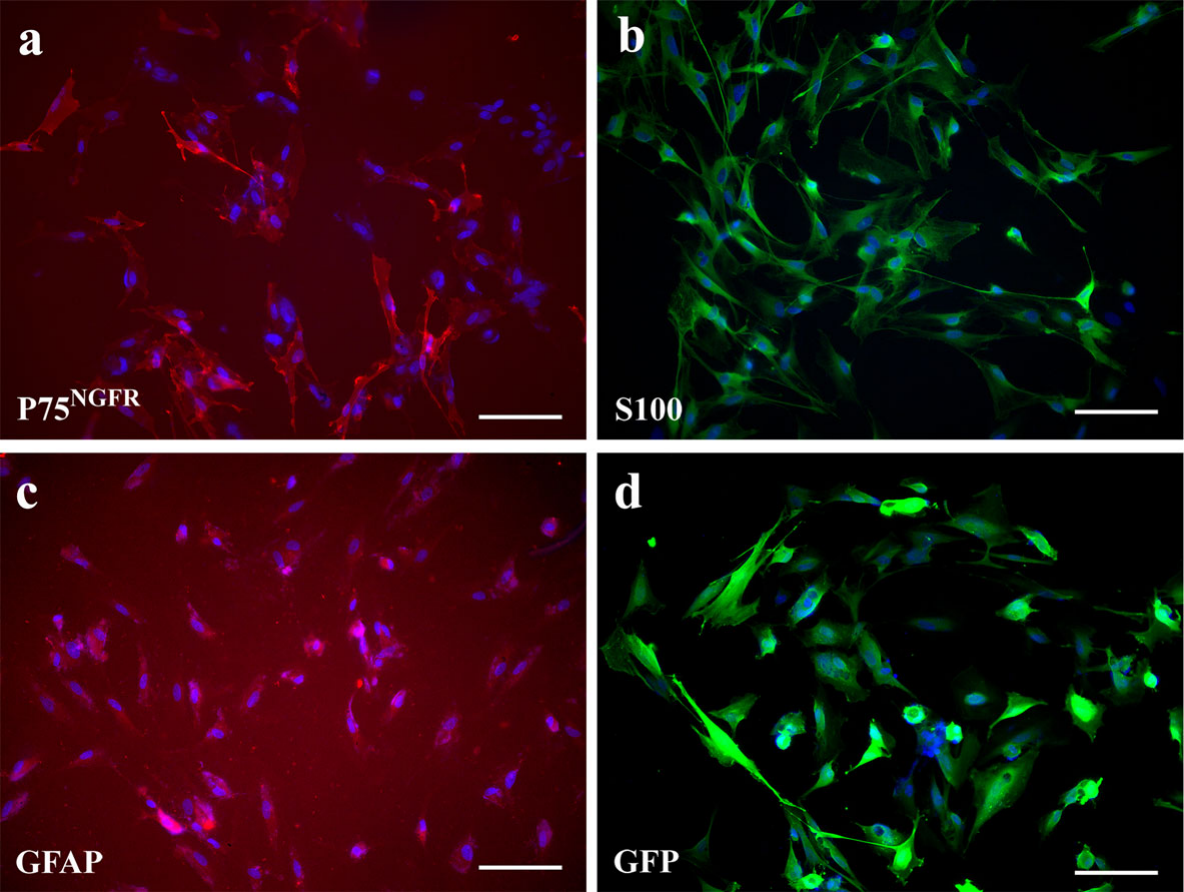
Characterization of cultured olfactory ensheathing cells (OEC). **a**: low affinity nerve growth factor receptor (p75^NGFR^) immunoreactivity of OEC, (**b**): S100 immunoreactivity of OEC, (**c**): GFAP immunoreactivity of OEC, (**d**): OEC expressing GFP at 3 days after Ad-GFP infection. p75^NGFR^(+) cells 90.8 ± 4%; S100(+) cells, 91.3 ± 2.9%; weak GFAP (+) cells, 97.0 ± 1.3%. Nuclei are counterstained with Hoechst 33258 (blue). Bar = 50 μm. Data are expressed as average ± SD and represent the results of two independent experiments performed in triplicate (n = 6 per experimental conditions). Note that OEC are highly permissive to Ad-GFP infection

We have successfully established OEC culture from adult rat olfactory bulb. After grown to confluence, OEC were subcultured and processed for immunocytochemistry. Histologic analysis of cultured OEC revealed that >90% of cells were immunoreactive to low affinity nerve growth factor receptor (p75), S100 and GFAP (Fig. 1a-c, 90.8 ± 4%, 91.3 ± 2.9%, 97.0 ± 1.3%, respectively, compared to Hoechst 33258(+) cells). The infective tropism of recombinant adenovirus in OEC was test using AdGFP infection. AdGFP was added to OEC culture with 50 MOI, resulting in transduction of virtually all cultured cells (Fig. 1d). This indicates that OEC are highly permissive to AdGFP infection.

**Fig. 2:**
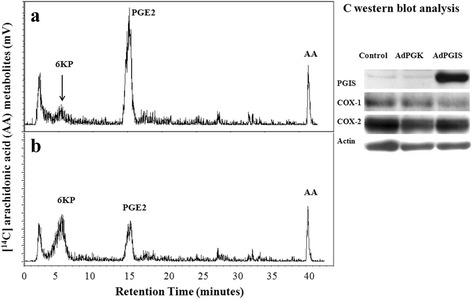
The enzyme activity and respective protein expression of eicosanoid biosynthesis in cultured OEC. **a** & **b**: HPLC analysis of ^14^C-labelled eicosanoids generated in Control and AdPGIS-transduced OEC cultures, respectively, in response to [1-^14^C] arachidonic acid (AA). 6-KP denotes 6-keto-PGF_1α_, the product of PGI_2_ hydrolysis. Each prostanoid peak was verified by coelution with an authentic radiolabelled prostanoid, **c**: Western blot analysis of protein levels of PGIS, COX-1 and COX-2 in cultured OEC receiving AdPGK or AdPGIS infection. Measurement of eicosanoid biosynthesis and biosynthetic enzyme expression in cultures was conducted at 3 days after Ads transduction. Confluent OEC cultures were pulsed with [1-^14^C] AA for 10 min. The released [1-^14^C] eicosanoids in the released medium of OEC were purified and subsequently analyzed by HPLC with a radiochemical flow-through detector. Note that OEC constitutively expressed COX-1 and COX-2 but not PGIS. On Ad-PGIS infection, PGIS expression was increased and AA metabolites in OEC were shunted through 6-ketoPGF_1α_ (6-KP) synthesis

Our pilot study has revealed the expressional existence of COX-1 and COX-2, but not PGIS, in cultured OEC. Thus, recombinant AdPGIS was chosen to infect cultured OEC for selective eicosanoid production. Recombinant AdPGK was used as a (mock) vector control. At 2–3 days after AdPGK or AdPGIS infection, the biosynthetic activity of eicosanoid in confluent OEC cultures was analyzed. Fig. 2a & b shows the HPLC spectrum of ^14^C-labelled eicosanoid production by cultured OEC in response to [1-^14^C] arachidonic acid (AA) treatment. There were two predominant peaks, ^14^C-labelled prostaglandin (PG) E_2_ and AA in nontreated OEC cultures (Fig. 2a). Very small peak of prostacyclin, shown as its hydrolysis product 6-keto- PGF_1α_ (6KP), could be found in OEC. The transduction of AdPGK in cultured OEC did not alter the metabolic profile of [1-^14^C] AA (data not shown). Interestingly, AA metabolites were shunted through prostacyclin synthesis on AdPGIS transduction to OEC. An increased 6KP level with a concurrent decrease of PGE_2_ level was demonstrated in AdPGIS-transduced OEC cultures (Fig. 2b). This indicates that the overexpressing PGIS in OEC was functionally active in producing prostacyclin from AA. Western blot analysis of OEC further revealed that OEC expressed high levels of COX-1 and COX-2 but not PGIS. On AdPGIS infection, expression of PGIS was increased (Fig. 2c).

**Fig. 3:**
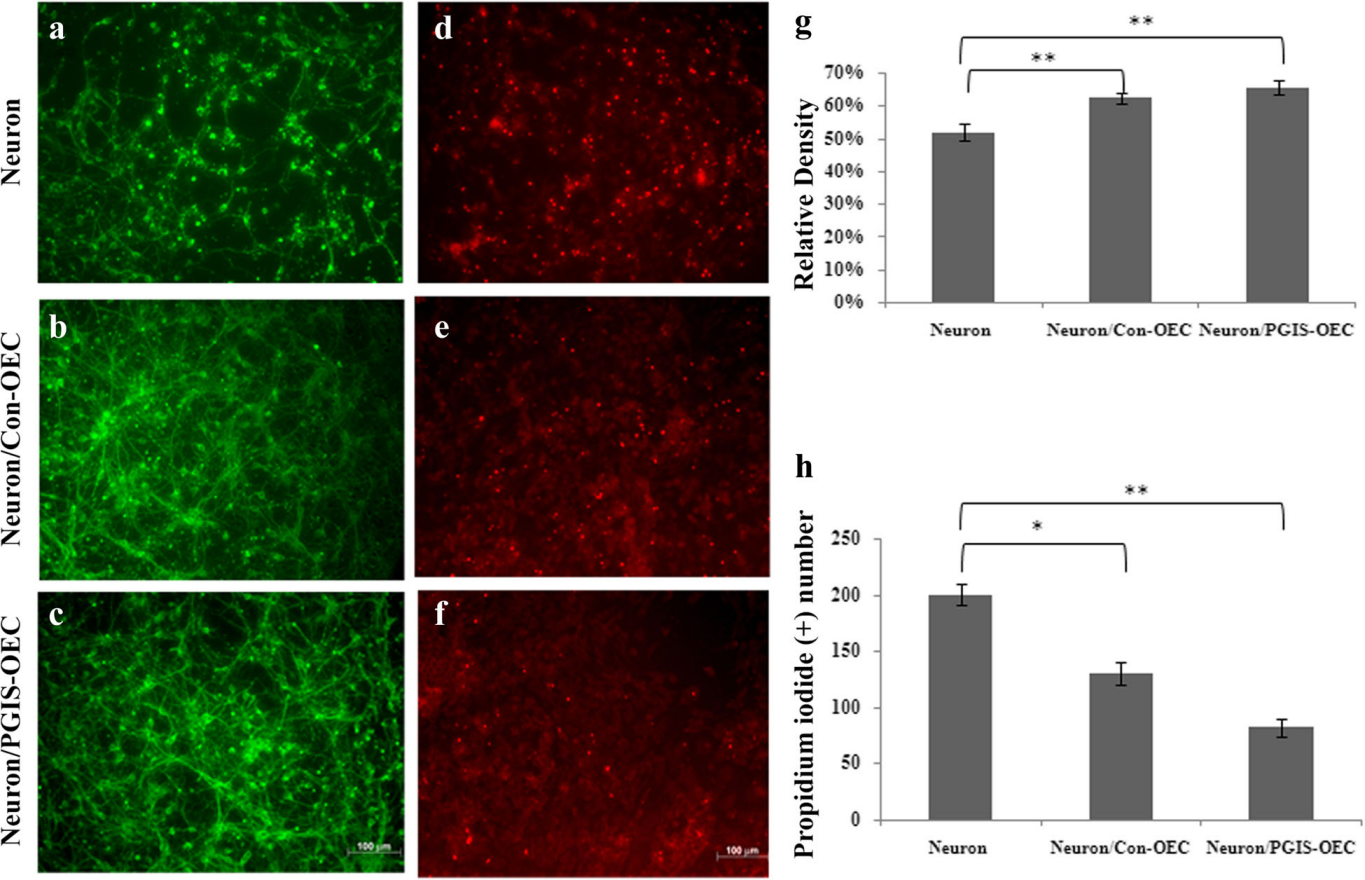
OEC co-cultures protected OGD-induced cell damage in mixed neuron-glial cultures. **a** & **d**: neuron-glial cultures, (**b** & **e**): Con-OEC cocultured with neuron-glial culture, (**c** & **f**): PGIS-OEC cocultured with neuron-glial cultures, (**g**): quantitative bar chart for tubulin-IR density, (**h**): quantitative bar chart for PI (+) cell numbers in neuron-glial cultures. **a**-**c** are tubulin immunoreactive cells, (**d**-**f**) are PI (+) cells. Bar = 100 μm. *,** *P* < 0.01,0.05, compared to neuron-glial cultures. One-way analysis of variance + Bonferroni post test. Data were expressed as mean ± SEM, represent the results of three independent experiments (*n* =8-10 per experimental conditions)

**Fig. 4:**
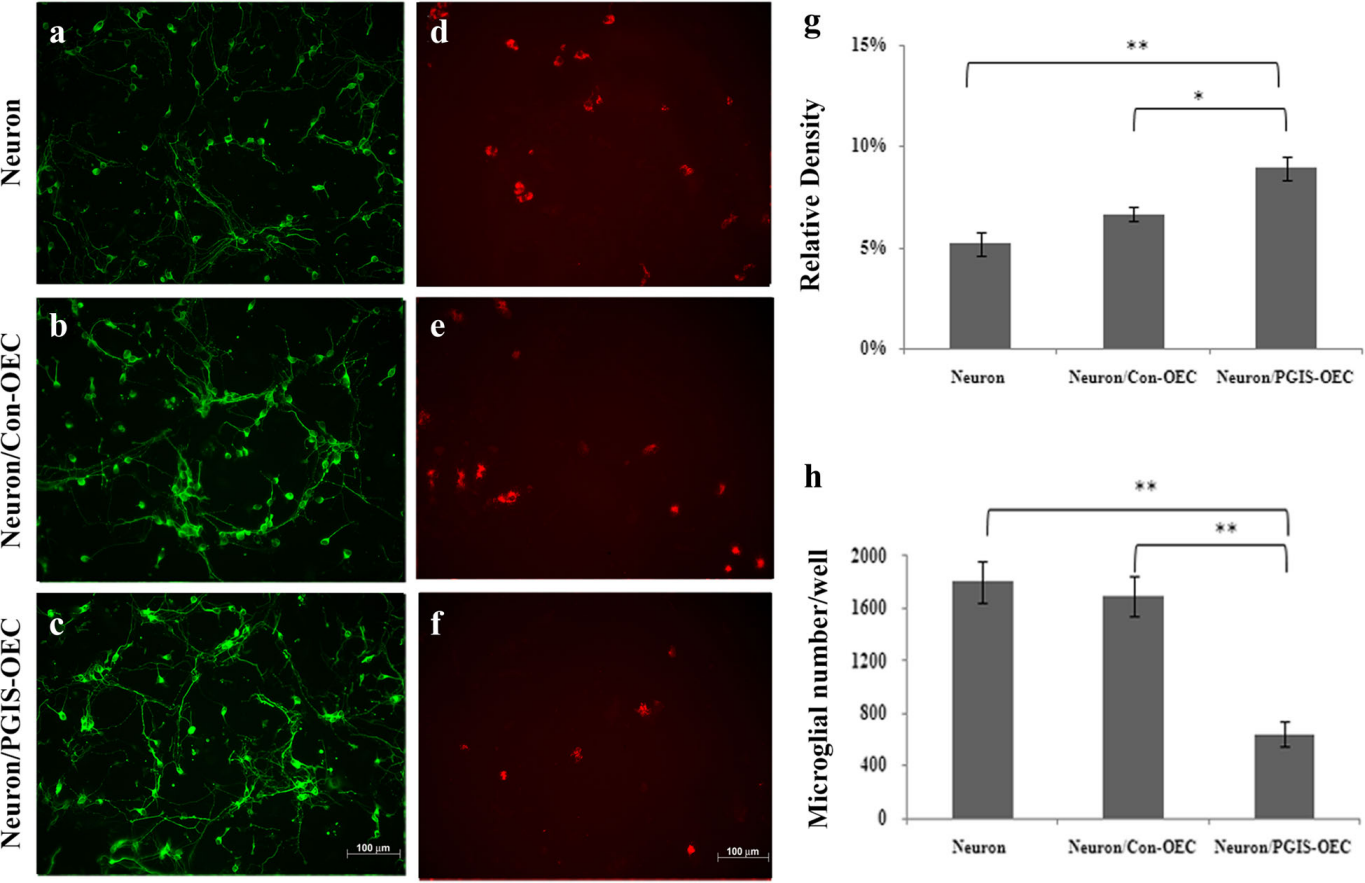
PGIS-overexpressing OEC cocultures enhanced neuronal connection and reduced microglial number in spinal cord neuron-glial cultures. (**a** & **d**): neuron-glial cultures only, (**b** & **e**): neuron-glial cultures cocultured with Con-OEC, (**c** & **f**): neuron-glial cultures cocultured with PGIS-OEC, (**g** & **h**): quantitative bar charts for tubulin immunoreactivity and ED1 (+) cell numbers, respectively. A-C are tubulin immunoreactive cells and D-F are ED1 (+) microglial cells. Bar = 100 μm. *,** *P* < 0.01,0.05, compared to neuron-glial cultures, by one-way analysis of variance + Bonferroni post test. Data were expressed as mean ± SEM, represent the results of two independent experiments performed in duplicate-triplicate

Beneficial effect of OEC or PGIS-overexpressing OEC (PGIS-OEC) was first examined by co-culture of OEC with cortical neuron-glial cultures after treatment with OGD, an in vitro ischemia. Neuron-glial cultures underwent 3 h of OGD stress and returned to normoxic condition. OEC or PGIS-OEC (seeded in Millicell insert well) was then transferred to these stressed neuron-glial culture and further incubated for 2 days. Fig. 3a-c shows that the stressed neurons survived much better, as demonstrated by tubulin-IR, in both OEC and PGIS-OEC co-cultures, compared to neuron-glial cultures only. The relative density of tubulin-IR in both co-cultures were significantly higher than those in neuron-glial cultures only (Fig. 3g; *P* < 0.01). However, the neuronal densities between these two OEC-treated groups show no difference (Fig. 3g). Concurrently, PI (+) cells in both OEC co-culture groups were significantly less than those in neuron-glial cultures only (Fig. 3d-f and h; *P* < 0.01 or 0.05). This result indicated that OEC cocultures effectively protected neuronal cells from OGD-induced damage. We then examined the effect of OEC or PGIS-OEC insert on neuron or microglia in spinal cord neuron-glial cultures. As shown in Fig. 4a-c & g, neuronal connection (tubulin-IR density) was significantly better in PGIS-OEC co-cultures, compared to that in neuron-glial cultures only or its co-culture with OEC (*P* < 0.05 or 0.01). By contrast, ED1-IR-microglial number in neuron-glial cultures was significantly reduced by PGIS-OEC co-cultures, compared to that in neuron-glial cultures only or by OEC co-culture (*P* < 0.01) (Fig. 4d-f & h).

**Fig. 5:**
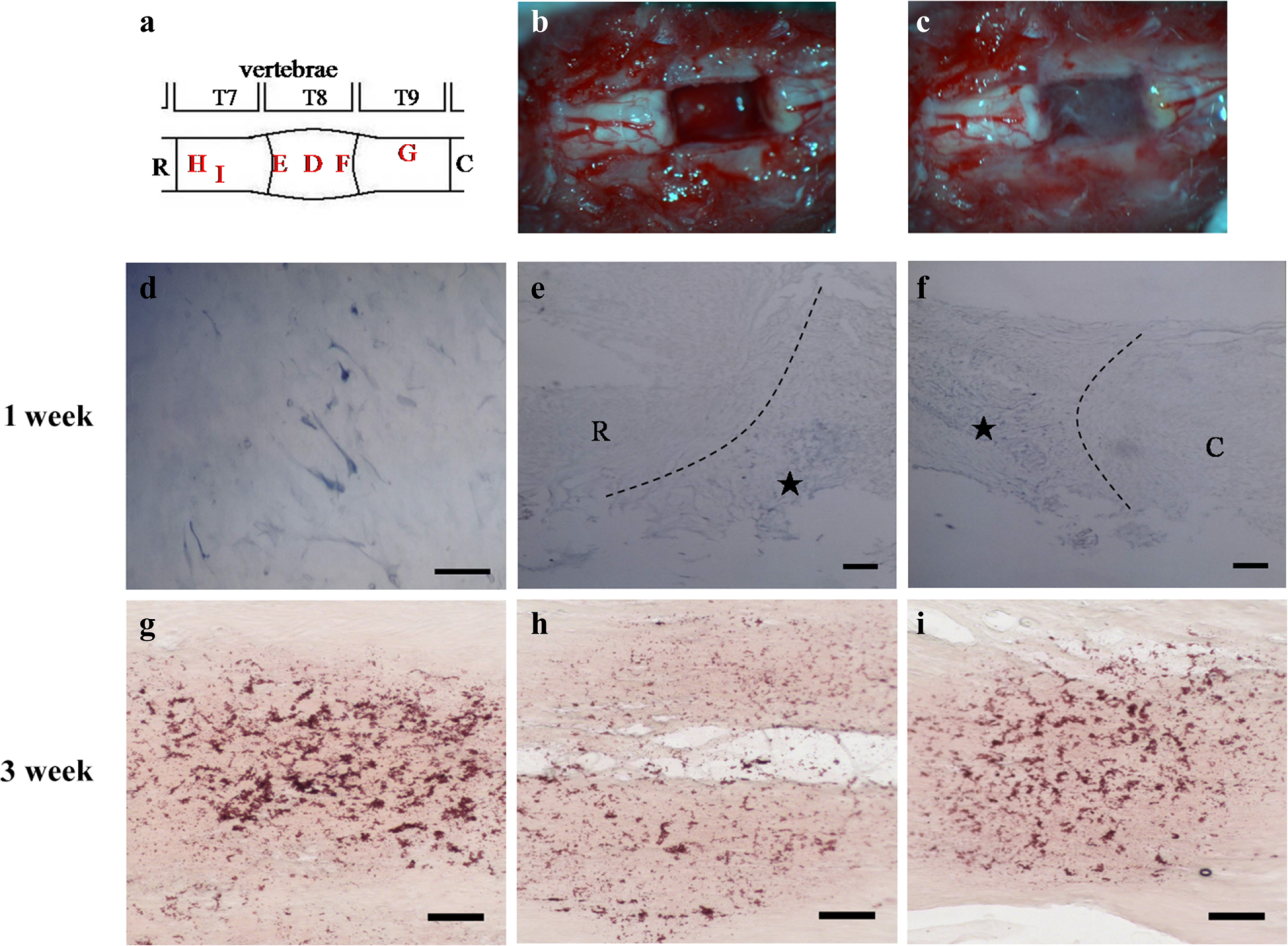
Spinal cord transection injury and transplantation of OEC **a**: Scheme showing segments of thoracic spinal cord (T7 ~ T9; R: rostral; C: caudal). The location of GFP expressing OEC was depicted (EDF), **b** Complete transection injury (with a 5 mm gap) in thoracic spinal cord, **c**: Transplantation of fibrin glue-mixed OEC to the transection cavity, **d**-**f** GFP-expressing OEC in the transection cavity of spinal cord at 1 week post-transplantation. **d** Higher magnification of GFP (+) OEC. Bar = 150 μm in d, 50 μm in e&f. **g**-**i** GFP-expressing OEC in the spinal stumps of 3 week SCI rats at 4–5 mm rostral or caudal to the lesion epicenter. Bar = 50 μm. Adult OEC were prelabelled with GFP by adenovirus- mediated gene transfer before transplantation. Note: At one week post-transplanation, GFP (+) OEC were found in the transected cavity (in **d**) and in regions close to both spinal stumps (marked with star in **e** & **f**). At 3 weeks postgraft, GFP (+) OEC migrated to both stumps at 4–5 mm rotstral or caudal to the lesion epicenter. Grafted AdGFP-OEC could survive and express GFP persistently in the host spinal cord for at least 3 weeks

The in vivo growth-promoting properties of OEC or PGIS-OEC were demonstrated by transplantation of each OEC to the complete T8-transected cavities of rat spinal cords. Adult OEC were pre-transduced with GFP or PGIS by adenovirus-mediated gene transfer. AdGFP- or AdPGIS-infected OEC in fibrin solution were then applied to the transected cavity (5 mm gap) of spinal cord at thoracic level 8, as shown in Fig. 5a-c. Figure. 5b shows a complete transection of T8 with a 5 mm gap. Fig. 5c shows fibrin-mixed OEC was grafted to the transected T8 gap. To trace OEC graft in vivo, the AdGFP-infected OEC transplanted SCI rats were sacrificed respectively on the first, third, fifth, eighth and twelfth week after surgery. Fig. 5d-f shows GFP-immunoreactive OEC in the longitudinal spinal section (T7 ~ T9) of AdGFP-infected OEC grafted SCI rats. At one week postgraft, GFP (+) OEC were found in the transected cavity (Fig. 5d) and in regions close to both spinal stumps (marked with stars in Fig. 5e & f), and the cells did not migrate into the rostral/caudal stumps of the spinal cord. We could not find any GFP (+) OEC in the grafted site at third weeks post-graft onwards in SCI rats. Grafted OEC might migrate into spinal cord stumps. As expected, GFP (+) OEC was found in both rostral/caudal stumps of the spinal cord about 4–5 mm away from the injured epicenter at third weeks post-graft (Fig. 5g-i).

**Fig. 6:**
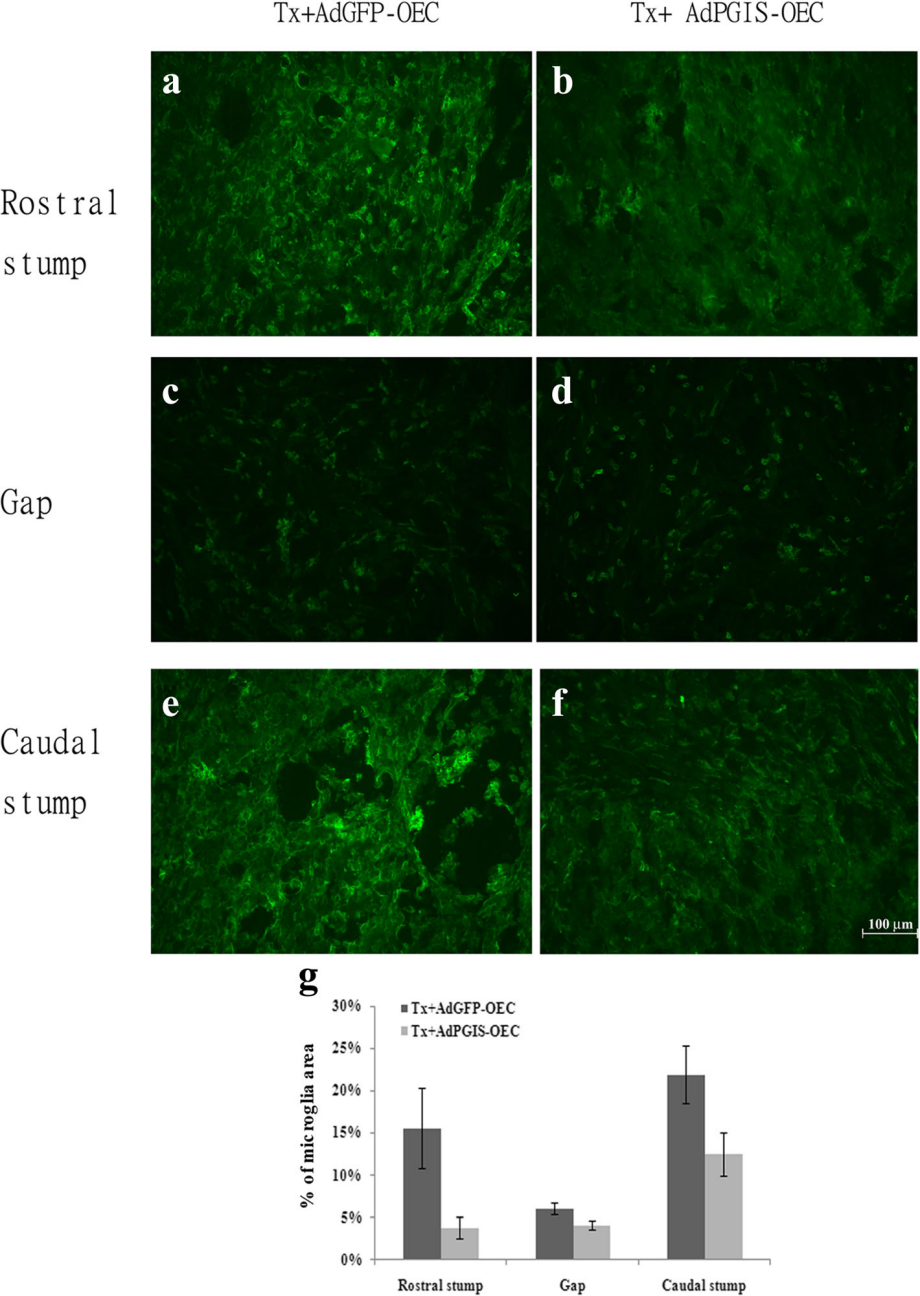
Iba1-immunoreative microglial cells in the spinal cords of SCI rats at 3 weeks postinjury. **a** & **b**: rostral stump; **c** & **d**: transected gap; **e** & **f**: caudal stumps. **a**, **c**, **e**: GFP-OEC-grafted spinal cords; **b**, **d**, **f**: PGIS-OEC-grafted spinal cords. Green label shows Iba1 positive microglia. **g**: The histograms show the percentage of Iba1-positive microglia in the spinal cords of SCI rats

To examine the effects of prostacyclin on inflammatory cells, Iba1-immunoreactive microglia in the 3 week injured spinal cords of the AdGFP-OEC-grafted and AdPGIS-OEC-grafted groups were compared. As shown in Fig. 6 Iba1 immunoreactivity was most prominent in the rostral/caudal stumps of two groups, whereas relative few Iba1 immunoreactivity was found in the transection gap. Microglial cells in the rostral/caudal stumps of AdPGIS-OEC grafted spinal cords have less immunoreactivity than that of AdGFP-OEC grafted spinal cords. Whereas in transected gap, microglial cells have similar immunocreactivity between AdPGIS-OEC-grafted and AdGFP-OEC-grafted spinal cords.

**Fig. 7:**
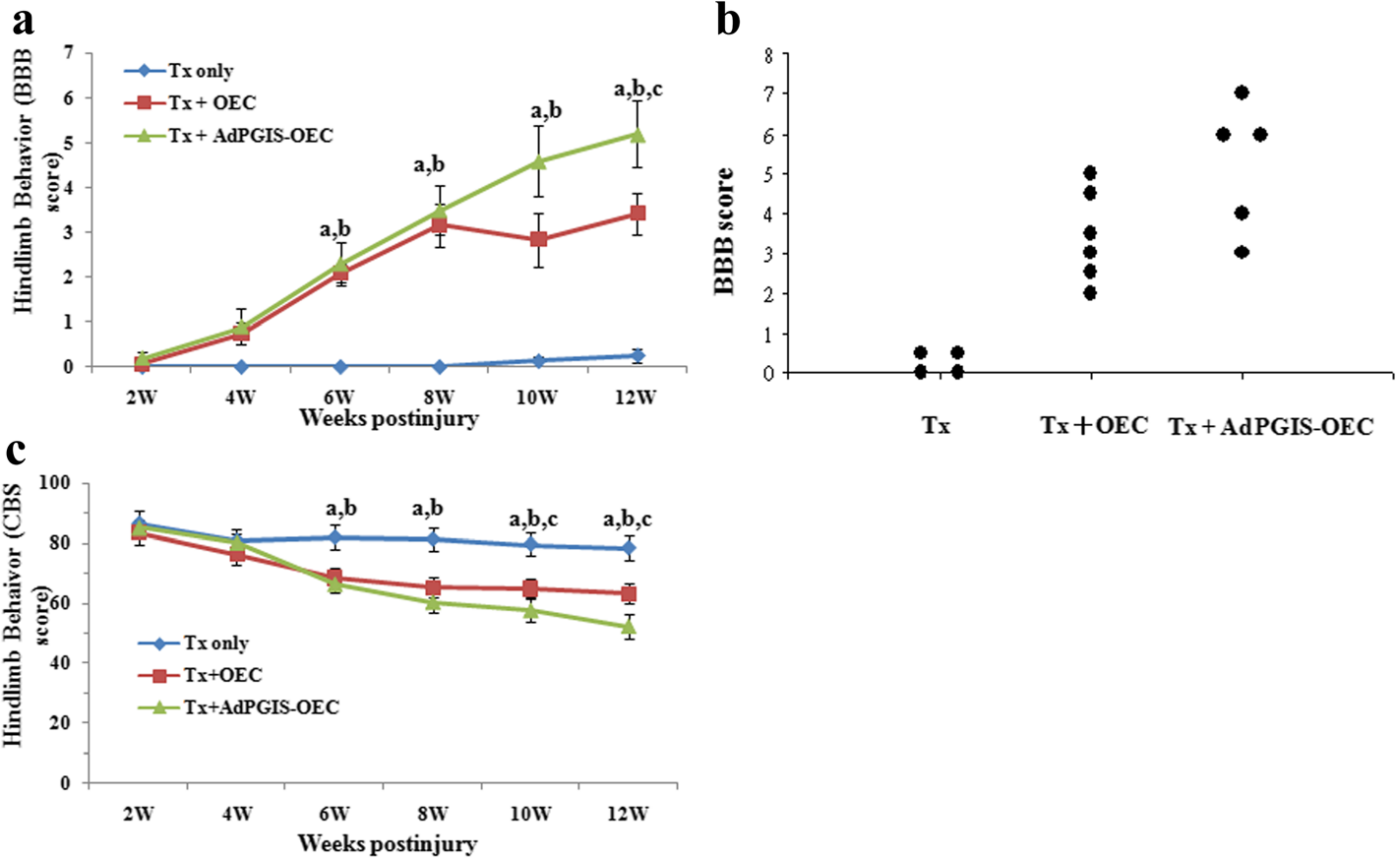
Improving hindlimb locomotor functions in SCI rats receiving OEC or AdPGIS-OEC transplant after transection spinal cord injury. **a** & **b**: BBB scores, expressed as line chart and scatter plot, respectively, of SCI rats receiving none, OEC graft or AdPGIS-OEC graft, (**c**): CBS score of SCI rats receiving none, OEC graft or AdPGIS-OEC graft. OEC transplant induced functional recovery after a complete transection in spinal cord. Scatter plot showing the BBB score for the control (TX) and transplant animals at 12 weeks after the transplantation of OEC. **a**, *P* < 0.05 Tx + OEC Tp versus Tx only;b, *P* < 0.05 Tx + AdPGIS-OEC Tp versus Tx only; **c**, *P* < 0.05Tx + AdPGIS-OEC Tp versus Tx + OEC Tp。Data were analyzed by ANOVA followed by LSD test. Data represent the mean ± SEM

To ensure all experimental rats receiving similar degrees of injury and to monitor the rate of their recovery, the rats were subjected to hindlimb locomotor functional assessment by BBB and CBS scores. As shown in Fig. 7a, OEC transplant induced functional recovery in rats after a complete transection injury in spinal cord. The BBB scores of the SCI rats receiving OEC and AdPGIS-OEC were higher than that in SCI rats with vehicle treatment (BBB scores 2 –5). The values were significantly different from those observed in SCI rats with vehicle treatment (BBB scores <1). The BBB scores in vehicle-treated rats were similar to those shown by our previous studies [1, 2, 39]. Starting from 6 weeks through 12 weeks post-graft, OEC or AdPGIS-OEC treatment significantly improved the hindlimb function of SCI rats (all *P* < 0.05). The BBB scores of OEC or AdPGIS-OEC were not different from each other until 12 week post graft (*P* < 0.05). The locomotion in SCI rats receiving AdPGIS-OEC was improved at 12 week post SCI with significant increase of BBB score (BBB:5.2 ± 0.7, Fig. 7a) over that in OEC graft only (BBB:3.4 ± 0.5). Fig. 7b reveals the scatter plot of BBB scores of each SCI rat receiving none, OEC graft or AdPGIS-OEC graft at 12 weeks post treatment. Fig. 7c shows CBS scores of these experimental rats. Compared to non-treated group, the experimental groups that received OEC or AdPGIS-OEC transplantation achieved better restoration. SCI rats with OEC or AdPGIS-OEC grafts achieved improvement (significantly lower CBS score) in combined behavior function than vehicle-treated SCI rats (*P* < 0.05). At 10 & 12 week postgraft, the CBS of AdPGIS-OEC grafted SCI rats were significantly different from that of OEC grafted SCI rats (*P* < 0.05). However, none of the animals demonstrated coordinated movements of fore and hind limbs or the ability to bear weight on the hind limbs.

**Fig. 8:**
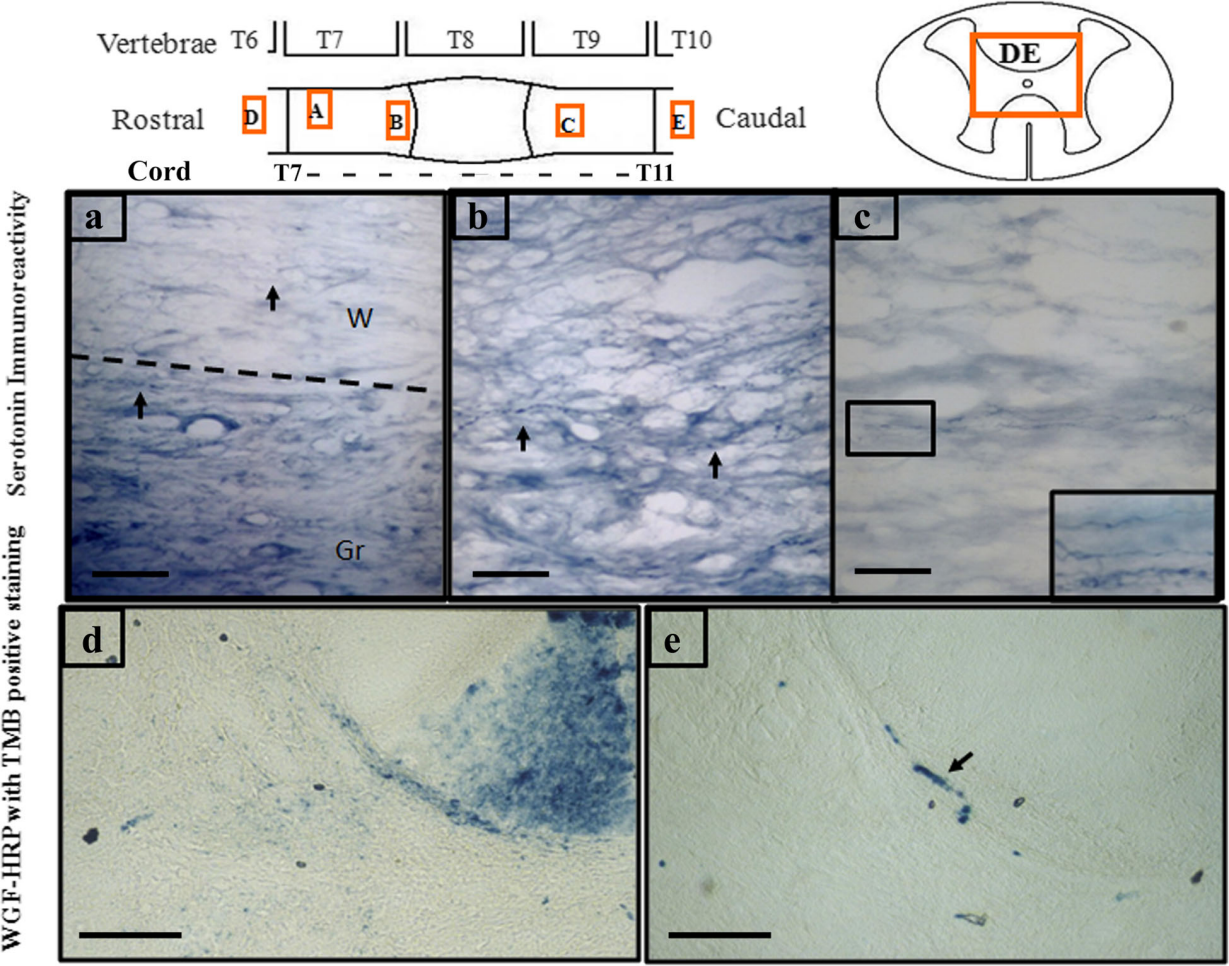
Serotonin immunoreactive nerve fibers and WGA-HRP labelled signals were detected in the proximal and distal stumps of AdPGIS-transduced OEC-transplanted spinal cords. **a**-**c**: Serotonin immunoreactivity in the longitudinal section of spinal cord. Bar = 50 μm. **d** & **e**: WGA-HRP labelled signals with TMB positive staining in the proximal and distal spinal cords, respectively, of animals grafted with AdPGIS-OEC using anterograde WGA-HRP tracing from motor sensory cortex. Bar = 100 μm. a and b are longitudinal sections of proximal cord. The dotted line indicate the separation between grey and white matter in panel **a**. Arrow indicate 5-HT(+) fibers. c shows the 5-HT(+) fibers in the gray matter of OEC-transplanted distal cords (longitudinal section). Inset: higher magnification of boxed inset in (**c**). **d** and **e** are transverse sections of T7 (proximal) and T12 (distal) stumps, respectively

At 12 weeks post injury and treatment, rats were subjected to histological analysis or anterograde tracing for assessing neural regeneration. Fig. 8a-b shows typical serotonin immunoreactive fibers in the proximal stump of OEC- or AdPGIS-OEC-grafted spinal cord. Fig. 8c shows a few regenerating 5-HT fibers in the distal stump of OEC-grafted spinal cord. Such serotonergic axons in the distal cord were observed in one out of six OEC-transplanted SCI rats (16.7%) and three out of five AdPGIS-OEC transplanted SCI rats (60%). Regarding the WGA anterograde tracing results, Fig. 8d demonstrates the TMB (+) staining fibers of corticospinal tract in the (intact, thoracic T7) proximal stump of AdPGIS-OEC-grafted spinal cord. A few regenerating TMB (+) staining (arrow) fibers were found in the distal stump (at thoracic T12; Fig. 8e) of two out of six AdPGIS-OEC-graft SCI rats (40%). Nothing could be found in the distal stump of transected SCI rats. Table 1 reveals the corresponding results of animal behavior, neuronal tracing of WGA-HRP and serotonin immunoreactivity at 12 weeks after injury and treatment.

**Table 1:** Results of animal behavior, neuronal tracing of WGA-HRP and 5-TH immunoreactivity at 12 weeks after injury and treatment

Rats	WGA-HRP tracing		5-HT	Behavioral test (12 W)	
	Anterograde	Retrograde		BBB	CBS
Tx only					
1	-		-	0	80
2		-	-	0	80.5
3	-		-	0.5	77.5
4			-	0.5	76
	0%		0%		
Tx + OEC Tp					
5	-		-	5	63
6		-	-	2.5	65
7	-		-	4.5	60
8			+	3	64.5
9	-		-	2	64.5
10	-		-	3.5	63
	0%		16.7%		
Tx + AdPGIS-OEC Tp					
11	+ (T12)		+	7	44
12	-		+	6	46.5
13	-		-	4	59
14	+ (T12)		+	6	46
15		-	-	3	66
	40%		60%		

A few 5HT (+) fibers were found in the distal stump, within 1 mm distal to the transected gap, in one Tx + OEC Tp and three Tx + AdPGIS-OEC Tp rats

Anterograde tracing was conducted from motor cortex in experimental rats. WGA (+) fibers were found in the thoracic level 12 in two Tx + AdPGIS-OEC Tp rats. Whereas retrograde tracing was conducted from T12, no WGA (+) signals were found in the motor cortex of SCI rats (one rat per group)

## Discussion

The central observation of this study is that overexpression of prostacyclin synthetase in OEC mediated through Ad gene transfer selectively enhanced prostacyclin synthesis and concurrently reduced other eicosanoid levels. This PGIS overexpressing OEC had good neuroregenerative potential in cortical or spinal neuron-glial cultures. Further in vivo studies showed that transplantation of OEC to the injured site in SCI rats facilitated axonal regeneration across a 5 mm transection gap and improved hindlimb function. Transplantation of AdPGIS-transduced OEC to T8-transected rats further promoted fiber tract regeneration and significantly improved the functional outcome. We have shown evidence of axonal regeneration across a complete spinal cord transection in 40% of AdPGIS-OEC spinal rats by anterograde WGA tracing from motor sensory cortex. Regenerating serotonergic axons in the distal cord were also observed in one of OEC-transplanted rats (16.7%) and three of AdPGIS-OEC transplanted rats (60%). Nothing was found in the transected SCI rats.

We have established adult OEC cultures by their unique seeding properties to plastic dish. More than 90% of these cells were immunoreactive to S100 and p75, the low affinity nerve growth receptor. This is consistent with the reported characteristic of OEC, a specialized glia with both Schwann cell-like and astrocyte-like properties [17, 34, 42]. We further found that cultured OEC actively metabolized exogenous arachidonic acid into PGE_2_, a COX-1- or COX-2-derived product. The high AA metabolic activity of OEC is quite similar to that of astrocytes reported in our recent article [29]. However, this is in contrast to low AA metabolic activity in neuron-glial cells, which released barely detected eicosanoids [28]. Interestingly, gene transfer of AdPGIS to OEC shunted the AA catabolism through synthesis of 6-keto-PGF_1α_, a hydrolyzed product of prostacyclin and the production of other eicosanoids was concurrently reduced (Fig. 2). This indicates that the overexpressing PGIS was functionally active in cooperation with endogenous COX to produce prostacyclin. Prostacyclin is the most potent inhibitor of platelet aggregation and a cytoprotective agent against various stresses [21, 22]. However, it is very unstable with biological half-life time being only 2–3 min. Prostacyclin could also dilate the blood vessels, and inhibit the activity of white blood cell and monocyte in the inflammatory reaction. Many studies showed cytoprotective effects of prostacyclin, but the mechanism was still not yet clear. Cytoprotective effects of prostacyclin could include the function of removing reactive oxygen species [43]. Prostacyclin had certain protective function for spinal cord injury partly because it could resist other PGs, which had the function of vasoconstriction and platelet aggregation [31, 44]. Prostacyclin could inhibit inflammatory cells from aggregating in the injured tissues and alleviated local inflammatory reaction [28, 29].

The functional characterization of OEC was examined by in vitro co-culture with neuron-glial cultures. A clear neuroprotective effect of OEC was observed on OGD-treated cortical neuron-glial cells. Both control OEC and PGIS-OEC effectively reduced cell death and enhanced neuronal survival. Such properties of OECs might render them potential clinical agents able to support injured CNS. In co-culture with spinal cord neuron-glial cultures, PGIS-OEC enhanced neuronal connection significantly better than OEC. By contrast, microglial number in neuron-glial cultures was significantly reduced by PGIS-OEC co-cultures. In three week PGIS-OEC grafted spinal cords, microglial numbers were reduced compared to those in GFP-OEC grafted spinal cords (Fig. 6), consistent with co-culture study and our previous report [28]. The prostacyclin receptor (IP receptor) is a G protein–coupled receptor that is primarily coupled to the activation of adenylate cyclase, which catalyzes the formation of 3′,5′ cyclic adenosine monophosphate (cAMP), a key messenger for axon regeneration. The benificial effect of PGIS-OEC in co-cultures or in grafted spinal cord may be, in part, mediated through increase of celllar cAMP. Reducing microglial activation/infiltration by prostacyclin is another possible effect because microglia plays a pivotal role in inflammatory reaction and the suggested effects from our previous reports [28, 29]. However, it remains to be determined which receptor subtype of prostacyclin (IP1 or IP2) is involved in these effects. Further studies are needed to elucidate the role played by downstream molecules in the inhibition of microglial number/activation.

After adult rat suffered from spinal cord injury, 5-10% spared fibers at the lesion center could make partially or fully functional recovery in the paralyzed hind limbs in rats [45]. Therefore, we chose the model of severe SCI with complete transection to evaluate the effect of the experimental therapy. This study ascertained that spinal cord was completely transected with 5 mm segment of the spinal cord being removed. However, it was useless to inject OEC into stumps since the distance between two stumps was 5 mm. Fibrin glue could be used as a bridge to connect CNS tissue [46]. In the present study, fibrin glue was employed to enwrap the transplanted OEC and reconnect two spinal stumps. The OEC embedded in fibrin glue could help new regenerating axon re-grow into the distal stump of spinal cord. Previous studies had shown that OEC graft could migrate in the host spinal cord together with growing axon [17, 47]. In order to observe if the fibrin glue-mixed OEC could survive and migrate in the host spinal cord, we applied AdGFP gene transfer to OEC before cell grafting. The result showed that OEC expressing GFP could be found at one and three weeks after surgery. At one week postgraft, OEC distributed inside the transplanted site and the edge of the transplanted area, and the cells did not migrate into the rostral/caudal stumps of the spinal cord, consistent with the result of Ruitenberg [48]. Grafted OEC migrated into spinal cord stumps at three weeks postgraft. As expected, GFP (+) OEC was found in both rostral/caudal stumps of the spinal cord about 4–5 mm away from the injured epicenter at third weeks post-graft (Fig. 5g-i), agreed with our previous result [34]. It is also likely/possible that OEC in the injured epicenter underwent apoptosis while OEC that migrated into the rostral/caudal stumps survived because of a less hostile environment. The recombinant Ad we used was the first generation and the expression of the encoding gene was temporary. AdGFP or AdPGIS used in this study had been constructed with a phosphoglycerate kinase promoter that could drive transgene expression with less immune reaction. There were no significant side effects in SCI rats with Ad transduced cells. Acute SCI is a devastating event and a secondary degenerative process is initiated following acute SCI. The secondary cascade occurs over hours and days after the initial insults and leads to further neurological damage. Intervention to block secondary pathological cascade after injury can protect cells and preserve function. The grafted PGIS-OEC in the injured cords continuously overexpressed PGIS (up to 3 weeks) that increased prostacyclin synthesis and exerted its neuroprotective and anti-inflammatory effects.

In the present study, axon regeneration was measured in two axonal systems: (i) descending corticospinaltract (CST) visualized by anterograde axonal tract tracer WGA-HRP and (ii) descending serotonergic (5-HT) tract, visualized by immunohistochemistry. WGA-HRP was injected stereotaxically into the hindlimb area of the sensory motor cortex. The main pathway of controlling motor in spinal cord was originated from the corticospinal tracts of primary motor cortex. Caudal axons labeled by WGA-HRP could be observed at thoracic level 12, but not further, in two of the AdPGIS-OEC grafted animals (~40%). The WGA-HRP-labeled axons located in the original pathway, between gray matter and white matter. However, we could not find any regenerated axons located in the edge of white matter of spinal cord as demonstrated in the study of Ramon-Cueto et al. [16]. On the other hand, the decending serotonergic fibers mainly projected to dorsal horn, medicinerea and ventral horn in spinal cord [49]. It could coordinate sensory, motor and autonomic functions via serotonin receptors [48]. Particularly, the 5-HT neurons projecting to ventral horn was related to the functional recovery after spinal cord injury [50]. Raphe nuclei were the only source of serotonergic fibers. Thirty days after complete spinal cord transection, the caudal stumps of the spinal cord tissue were not immunoreactive to 5-HT [2]. Thus, immunohistochemistrical staining of 5-HT could be used to examine if serotonergic axons regenerated through the lesion site and reached the caudal stumps of the spinal cord. In the present study, regenerating 5-HT (+) fibers could be found in the distal stump of one (16.7%) of OEC-grafted and three (60%) of AdPGIS-OEC grafted SCI rats. In the non-treated SCI animals, there was no serotonergic axonal regeneration found in the distal stump (0%). In addition, all regenerating axons were present only in the gray matter of distal spinal cord because of more glial cells and myelin in the white matter, which was not hospitable for regeneration of injured nerve tracts. Compared to OEC graft study in Lu et al. [51] which showed 5-HT (+) axon in the distal spinal cord, from 3.6 mm to the farthest 6 mm, the 5-HT (+) axon found in the present study was located not exceeding 1 mm from the transected site, and the amount was not much. We infer that it was related to the different transection gap between two stumps in the experiment. If we add on the distance in between two stumps, then the growing distance of the axon would be similar.

BBB scale [40] was designed for evaluating the motor functional recovery level of the hind limbs in rats after SCI. The advantage of it was that it was easy to execute and the animals used in the experiment did not need to be trained in advance. The behavioral result observed in the present study showed that there was no difference between groups until the fourth week postinjury. Until the sixth week, the difference appeared between the groups with transplanted OEC or Ad-PGIS-OEC and the control group. We inferred that it was related to the transected interval (5 mm) between two stumps in the experiment. It would take about 4–6 weeks for the axon to grow across this interval so that the average BBB score would not exceed 1 point before 4 weeks. Until the sixth week, it then had significant progress. In addition, we found that none of the animals had coordinate movement between fore and hind limbs in this twelve-week behavior observation, and the hind limbs could not bear the weight. Ramon-Cueto et al. [16] injected OEC into two stumps of transected spinal cord and found that the function of hind limbs could continuously recover and even bear the weight after seven-month behavior observation. Even though the functional recovery had limit in this twelve-week study, the functional recovery could be much better if we could have longer survival observation period.

## Conclusions

In conclusion, here we present the first evidence that enhanced synthesis of prostacyclin in grafted OEC significantly promoted functional restoration and improved fiber tract regeneration in spinal cord injured rats. These results suggest an important potential of prostacyclin in enhancing OEC therapeutic properties that are relevant for neural transplant therapies.

## Abbreviations

OEC: Olfactory ensheathing cells
PBS: Phosphate buffered saline
SCI: Spinal cord injuries
